# Supplementary material for: Transcriptomic Insights into Mechanisms of Early Seed Maturation in the Garden Pea (Pisum sativum L.)
Source: Cells. 2020 Mar 23;9(3):779. doi: 10.3390/cells9030779 (PMC7140803; doi:10.3390/cells9030779)

Sprint−2,10(3)

Global Summary

%DE = 0.25

genes with fdr < 0.2 = 7039 (3516 + / 3523 −)

genes with fdr < 0.1 = 6694 (3355 + / 3339 −)

genes with fdr < 0.05 = 6363 (3190 + / 3173 −)

genes with fdr < 0.01 = 5667 (2872 + / 2795 −)

genes in genesets = 0

<FC> = 0

<t−score> = 0

<p−value> = 0

<fdr> = 0.75

Global Genelist

Rank	ID	log(FC)	p−value	fdr	Metagene	Description
1	TRINITY_DN3	0.06	[illegible]	[illegible]	[illegible]	
2	TRINITY_DN2	−0.06	[illegible]	[illegible]	[illegible]	
3	TRINITY_DN1	−0.22	[illegible]	[illegible]	[illegible]	
4	TRINITY_DN4	0.13	[illegible]	[illegible]	[illegible]	
5	TRINITY_DN4	0.04	[illegible]	[illegible]	[illegible]	
6	TRINITY_DN4	−0.15	[illegible]	[illegible]	[illegible]	
7	TRINITY_DN3	0.09	[illegible]	[illegible]	[illegible]	
8	TRINITY_DN3	0.07	[illegible]	[illegible]	[illegible]	
9	TRINITY_DN3	0.08	[illegible]	[illegible]	[illegible]	
10	TRINITY_DN3	−0.04	[illegible]	[illegible]	[illegible]	
11	TRINITY_DN3	−0.05	[illegible]	[illegible]	[illegible]	
12	TRINITY_DN3	−0.05	[illegible]	[illegible]	[illegible]	
13	TRINITY_DN3	−0.07	[illegible]	[illegible]	[illegible]	
14	TRINITY_DN1	−0.2	[illegible]	[illegible]	[illegible]	
15	TRINITY_DN1	−0.05	[illegible]	[illegible]	[illegible]	
16	TRINITY_DN1	0.34	[illegible]	[illegible]	[illegible]	
17	TRINITY_DN1	0.16	[illegible]	[illegible]	[illegible]	
18	TRINITY_DN1	0.08	[illegible]	[illegible]	[illegible]	
19	TRINITY_DN1	0.04	[illegible]	[illegible]	[illegible]	
20	TRINITY_DN1	0.66	[illegible]	[illegible]	[illegible]	

Portrait

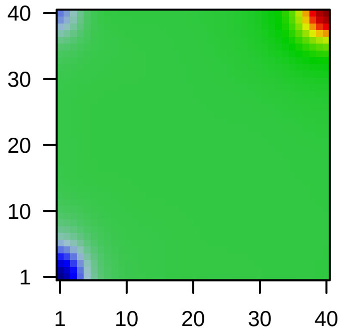

Top 100 DE genes

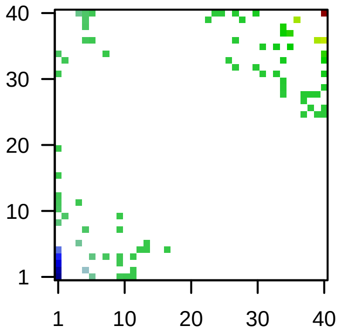

p−values

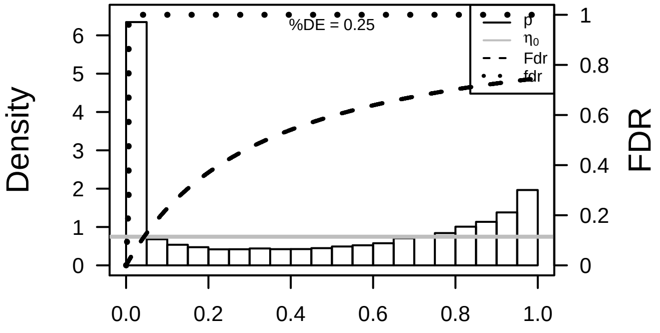

Supplement: Supplementary file 1 [file cells-09-00779-s001.zip › Supplementary materials/FileS2.zip!/FileS1/Summary Sheets - Samples/Sprint.2.10.3..pdf]
